# A geriatric assessment in general practice: prevalence, location, impact and doctor-patient perceptions of pain

**DOI:** 10.1186/s12875-016-0409-z

**Published:** 2016-01-28

**Authors:** Carsten Kruschinski, Birgitt Wiese, Marie-Luise Dierks, Eva Hummers-Pradier, Nils Schneider, Ulrike Junius-Walker

**Affiliations:** Institute of General Practice, Hannover Medical School, Carl-Neuberg-Str.1, 30625 Hannover, Germany; Institute of Epidemiology, Public Medicine and Healthcare Systems Research, Hannover Medical School, Hannover, Germany; Department of General Practice, University Medical Centre, Göttingen, Germany

**Keywords:** Pain, Aged, General practice, Geriatric assessment

## Abstract

**Background:**

To investigate what a geriatric assessment in general practice adds towards previous findings of prevalence, location, impact and the dyadic doctor-patient perception of pain in this age group.

**Methods:**

Cross-sectional study. Consecutive patients aged 70 and over underwent a comprehensive geriatric assessment in general practice that included a basic pain assessment (severity, sites and impact). Patients with pain and their doctors then independently rated its importance. Pain was correlated with further findings from the assessment, such as overall health, physical impairments, everyday function, falls, mood, health related lifestyle, social circumstances, using bivariate and multivariate statistics. Patient-doctor agreement on the importance of pain was calculated using *kappa* statistics.

**Results:**

219 out of 297 patients (73.7 %) reported pain at any location. Pain was generally located at multiple sites. It was most often present at the knee (33.9 %), the lumbar spine (33.5 %) as well as the hip (13.8 %) and correlated with specific impairments such as restrictions of daily living (knee) or sleep problems (spine). Patients with pain and their physicians poorly agreed on the importance of the pain problem.

**Conclusions:**

A basic pain assessment can identify older patients with pain in general practice. It has resulted in a high prevalence exceeding that determined by encounters in consultations. It has been shown that a geriatric assessment provides an opportunity to address pain in a way that is adapted to older patients’ needs – addressing all sites, its specific impact on life, and the patients’ perceived importance of pain. Since there is little doctor-patient agreement, this seems a valuable strategy to optimize concrete treatment decisions and patient centered care.

**Trial registration:**

This study is registered in the German Clinical Trial Register (DRKS00000792)

## Background

Pain is a central health issue for the majority of older people. It is the number one trigger for contacting a general practitioner (GP) in older patients [1], and constitutes about a quarter of all reasons for encounters [2]. However, it can be assumed that pain is not always actively reported, as in older patients it is one of many other relevant health problems and therefore is prone to be underreported in consultations. This has been suggested by a recent study, which found that a fifth of all patients with chronic pain are “silent sufferers” [3]. Moreover, poorly controlled pain impacts on older peoples’ health and lives, yet not much is known about pain related symptom and disease clusters or the doctor-patient dyadic perception of pain in primary care [4].

A geriatric assessment, as used for the present study, provides a good opportunity to examine pain in general practice. Its main purpose is to determine older peoples’ health in a standard way. It focuses on relevant and frequent conditions, such as impaired functions, limitations in daily activities, symptoms, mental and psychological state as well as on disease monitoring [5]. This diagnostic format has three practical advantages for a routine assessment of pain:

Firstly, it allows GPs to identify pain whether previously reported or yet “hidden”. Secondly, it facilitates the disclosure of pain at multiple locations. This is relevant, because the focus tends to be on single pain sites in the elderly [6], although multiple pain sites particularly impact on the progression of disability [7]. Thirdly, a geriatric assessment provides a comprehensive overview of older peoples’ health issues. It allows patients and doctors to weigh the relevance of pain in relation to concurrent health problems. This approach may come in useful, when treatment priorities need to be selected in the presence of multiple health problems [8].

As pain is usually not part of any routine assessment for older patients in German general practices, this paper intends to inform about the prevalence, location and impact of pain in general practice patients of 70 years and over, and to investigate and compare the relevance of pain from the patients’ and GPs’ views.

## Methods

### The main project

The present study is nested in the PräfCheck project (Check preferences: Proactive and shared treatment planning with older patients), study phase B [9]. The project was divided into a first part, in which a procedure was developed on how to select treatment priorities with older patients on the basis of the findings of a geriatric assessment (part A). This procedure incorporated a structured priority setting consultation using the principles of patient-centredness. In the second part B, the PräfCheck consultation was tested for effectiveness in the practice setting using a cluster-randomised controlled study design [10].

The procedure in part B was as follows: In the practices, study nurses performed a geriatric assessment with each participating patient. It included a basic pain assessment. Immediately afterwards, patients and their corresponding GPs independently rated each disclosed problem according to its deemed importance. This was done to create an awareness of the subjective relevance of concurrent patient problems. Whereas the control doctors merely discussed the assessment results with their patients in the following consultation, the intervention doctors additionally received the importance ratings of their patients, on the basis of which they performed a “PräfCheck” consultation to discuss priority problems [9, 10]. The study protocol was approved by the ethics committee of Hannover Medical School (No. 5069).

### Participating GPs and patients

Recruitment of GPs was based on zip codes. Initially, 43 GPs agreed to participate (response rate 18.6 %). In the practices, the reception staff consecutively approached every older patient who came to the office for whatever reason during a defined week. Inclusion criteria for patients were an age of 70 years or older and at least one contact with the GP in the previous three months. Exclusion criteria were long-term care dependency level II or III [9], dementia, limited contractual capability or incapacity, insufficient language skills, severe hearing loss, current participation in another clinical trial, and no availability by telephone. 347 patients gave their informed consent. Two doctors and 30 patients dropped out of the study for various reasons, before the assessment and the following importance ratings were completed. 10 patients did not provide all relevant data from the pain assessment, so that 297 patients of 41 GPs remained with full data sets.

### Geriatric assessment “STEP” and rating of health priorities

In this study the STEP instrument was used (Standardised Assessment for Elderly Patients in Primary Care). It was developed as a comprehensive assessment for older patients in general practice in an EU-Concerted Action. It consists of 76 single questions and two short performance tests characterizing 44 health and everyday problems [5]. STEP takes about 50 min to administer and has been tested and used in several trials [11–13]. The health priority rating took place after the STEP-assessment. Patients and physicians independently rated the importance of each disclosed problem. Patients assessed the importance of each problem once (importance for their life), whereas physicians rated the importance twice: (1) the importance for the patient’s medical care and (2) assuming the importance for the patient. For the present analysis, the ordinal response rating scale (from “1” = not important to “4” = very important) was dichotomized into “not important” or “important”. The patients’ importance ratings were compared to both types of the physicians’ ratings.

### Data used for the present study

For the present analysis, we used the data from the control and intervention participants. This included the data on all patient problems uncovered by STEP and the associated importance ratings by doctors and patients.

The pain assessment in STEP included a question that originates from the Medical Outcome Study (36-item Short-Form Instrument): “How much bodily pain have you had during the past 4 weeks?” (response options: none, mild, moderate, severe) [14]. Patients were also asked to estimate the extent to which pain limited their daily activities (response options: not at all, a little, fairly, very). The study nurses additionally marked each site of pain on a sketch of the body. For analysis, the body was partitioned into 32 (front) and 25 (back) sections. Only the sections in which at least 5.0 % of the patients reported pain were considered for the description of results, and only the three most frequent sites of pain were correlated with the variables described below. Further diagnostic questions that complete the assessment of pain, such as frequency, duration, character and triggers of pain, were not part of the standard geriatric assessment.

In order to correlate the presence of pain with other conditions uncovered by the assessment, we focussed on the following set of n = 14 variables from STEP. They were chosen as they may have an association with pain according to literature reviews and clinical experience. These included eleven dichotomous variables: presence of functional disabilities (basic activities of daily living [bADL], more complex instrumental ADL [iADL] and a general self-evaluation: “difficulty with usual activities, both inside and outside the house” [15], problem with home environment, physical symptoms and risks (sleeping disorder, two or more falls in the past six months, fractures after age of 60, depression, and not enough exercise). Gender and age group (< or ≥80 years) were considered as well as the overall subjective health status (first item of SF-36 on a scale of 1 = very good to 4 = bad) [14]. We added a variable on the total number of health problems as well as the number of different pain locations.

### Statistical analysis of the present study

Socio-demographic and pain data of patients were analysed descriptively. Then bivariate logistic regression analyses were performed to determine the association between the presence of pain at the three most frequent sites and each of the above mentioned 14 health parameters from the assessment. To further explore the independent effect of the 14 parameters taking their interaction into account, we conducted a stepwise forward logistic regression analysis using likelihood ratio tests for each pain site (multivariate model).

In order to determine the agreement between the importance ratings of patients and their GPs, we first established the observed frequencies of agreement. Then we calculated the chance-corrected agreement and employed the Cohen’s kappa co-efficient (κ). Values between 0 and 1 are interpreted in a range of agreement: 0.0-0.2, slight agreement; 0.21-0.4, fair agreement; 0.41-0.6, moderate agreement; 0.61-0.8, substantial agreement; and 0.81-1.0, nearly perfect agreement [16].

## Results

### Characteristics of patients and sites of pain

A total of 297 patients participated in the study, 219 of which (73.4 %) suffered from pain at any site. Patients were on average 77 years old. Age, educational status and income were not significantly different between the pain and non-pain group. In the pain group, however, there was a higher proportion of women (64.8 %) compared to the non-pain group (51.3 %; p < 0.001). The pain group also revealed a higher number of health problems and rated their health significantly worse compared to the non-pain group (see Table 1).

**Table 1:** Patient characteristics of the whole sample and related to presence or absence of pain

Patients	Total	Pain	No pain	*p*
	(N = 297)	(N = 219)	(N = 78)	
age (years)	77.0 (±5.0)	77.1 (±5.0)	76.9 (±4.9)	0.83
health problems (n)	11.2 (±4.6)	12.0 (±4.6)	9.0 (±3.8)	<0.001
self-rated health	2.4 (±0.7)	2.5 (±0.7)	2.1 (±0.7)	<0.001
1 to 4 (very good to bad)				
education	1.9 (±0.7)	1.9 (±0.5)	2.0 (±0.5)	0.10
1 to 3 (low to high)^a^				
income	1.9 (±0.5)	1.8 (±0.7)	2.0 (±0.8)	0.06
1 to 3 (low to high)^b^				

^a^ Education: low: lowest school leaving certificate at best and no vocational training; medium: apprenticeship, skilled worker; high: academic degree

^b^ Income: low:<1000€, high:>1500€

Patients with pain provided specifications about their pain sites. Pain at just one site was present in 33 % of patients. 44 % specified two or three pain locations, and 23 % four or more. The sites of pain of the 219 patients are presented in Table 2, if at least reported by 5.0 % of the patients. The three most frequent sites of pain were the front of the knee (33.9 %), the lumbar spine (33.5 %) and the front of the hip (13.8 %).

**Table 2:** Frequency of pain sites, if at least reported by 5.0 % of the n = 219 patients with pain

	Number	Percent
front		
knee	74	33.9
hip	30	13.8
lower leg	19	8.7
shoulder	18	8.3
dorsum of the foot	15	6.9
upper leg	14	6.4
wrist	13	6.0
abdomen (umbilical)	13	6.0
abdomen (lateral)	11	5.0
back		
lumbar spine	73	33.5
shoulder	19	8.7
thoracic spine	14	7.8
lower leg	16	7.3
bottom (lateral)	15	6.9
cervical spine	14	6.4
upper leg	14	6.4
nape of the neck	14	6.4
neck	12	5.5

### Association of patient characteristics with sites of pain (bivariate analysis)

Each of the selected 14 variables, covering patient characteristics and health conditions, was correlated with pain at the 3 most frequent sites. The results of these bivariate analyses are displayed as odds ratios (OR) and corresponding confidence intervals (CI) in Table 3. Knee pain was significantly associated with a larger number of patient characteristics in comparison to low back pain and hip pain. Some significant associations occurred at each of the three pain sites, in particular greater numbers of health problems and pain sites, and problems performing daily tasks. Three of the selected variables were not related to any pain site: age ≥ 80 years, fractures, and falls. Problems with bADL were much more present if the knee was the site of pain, whereas pain at the lumbar spine had higher odds of sleeping disorders than pain at the other sites. Reduced exercise was more often associated with either pain at the lumbar spine or the hip compared to the knee.

**Table 3:** Bivariate associations of pain for each of the 3 most frequent sites (knee, lumbar spine, hip)

	Patients without pain^a^ (%)	Patients with pain^a^ (%)		95 % CI		
			OR	Lower	Upper	*p* value
frontal knee (N)	223	74				
male gender	43.0	25.7	0.46	0.26	0.82	0.008
age ≥80 years	29.6	31.1	1.07	0.61	1.90	0.809
3+ pain sites^b^	1.76	3.07	5.49	3.12	9.66	<0.001
11+ health problems^b^	11.0	13.2	3.51	1.97	6.25	<0.001
poor subjective health	34.1	52.7	2.16	1.26	3.68	0.005
p.w. bADL	40.4	74.3	4.28	2.38	7.69	<0.001
p.w. iADL	10.3	31.1	3.92	2.04	7.55	<0.001
p.w. daily tasks	19.3	33.8	2.14	1.19	3.84	0.01
p.w.home environment	10.3	25.7	3.00	1.53	5.91	0.001
sleep disorder	33.2	47.3	1.81	1.06	3.09	0.03
depressed mood	28.7	44.6	2.00	1.16	3.44	0.01
fractures	22.4	20.3	0.88	0.46	1.68	0.70
falls	2.2	4.1	1.84	0.43	7.90	0.40
not enough exercise	29.6	37.8	1.45	0.84	2.51	0.19
lumbar spine (N)	224	73				
male gender	38.4	39.7	1.06	0.62	1.82	0.839
age ≥80 years	29.5	31.5	1.10	0.62	1.95	0.741
3+ pain sites^b^	1.44	3.26	4.11	2.36	7.18	<0.001
11+ health problems^b^	10.5	13.3	2.09	1.21	3.60	<0.001
poor subjective health	33.5	54.8	2.41	1.41	4.12	0.001
p.w. bADL	46.0	57.5	1.59	0.93	2.71	0.09
p.w. iADL	12.5	24.7	2.29	1.18	4.45	0.01
p.w. daily tasks	17.9	34.4	2.86	1.60	5.13	<0.001
p.w. home environment	12.1	20.5	1.89	0.94	2.78	0.07
sleep disorder	30.8	54.8	2.72	1.59	4.68	<0.001
depressed mood	30.4	39.7	1.51	0.87	2.62	0.14
fractures	21.4	23.3	1.11	0.59	2.09	0.74
falls	2.7	2.7	1.02	0.20	5.19	0.98
not enough exercise	27.7	43.8	2.04	1.18	3.53	0.01
frontal hip (N)	267	30				
male gender	39.0	36.7	0.91	0.42	1.98	0.808
age ≥80 years	31.1	20.0	0.55	0.22	1.41	0.209
3+ pain sites^b^	1.50	3.12	3.99	1.83	8.68	0.001
11+ health problems^b^	10.7	13.0	2.94	1.27	6.84	0.014
poor subjective health	37.1	53.3	1.94	0.91	4.14	0.087
p.w. bADL	46.4	70.0	2.69	1.19	6.09	0.01
p.w. iADL	14.6	23.3	1.78	0.72	4.43	0.21
p.w. daily tasks	21.0	40.0	2.51	1.14	5.52	0.02
p.w. home environment	13.5	20.0	1.60	0.61	4.19	0.33
sleep disorder	35.6	46.7	1.58	0.74	3.39	0.23
depressed mood	31.1	46.7	1.94	0.91	4.16	0.08
fractures	21.3	26.7	1.34	0.57	3.17	0.50
falls	2.2	6.7	3.12	0.60	16.13	0.16
not enough exercise	29.6	50.0	2.38	1.11	5.10	0.02

±14 parameters assessed as part of the comprehensive geriatric assessment that we hypothesized may have an association with pain

^a^ at particular location (knee or lumbar spine or hip)

^b^ 31 % of all patients had 3 or more pain sites, 51 % of all patients hat 11 or more health problems

*p.w.* problem with, *OR* Odds ratio for dichotomous predictor (present/absent), *CI* confidence interval

### Association of patient characteristics with sites of pain (multivariate analysis)

In multivariate analysis, some of the significant effects of the bivariate analyses were not sustained (see Table 4). Pain at a specific site obviously was not an isolated health problem as pain at any site was significantly associated with an overall greater number of pain sites. As already shown in bivariate analysis, pain at the knee was specifically associated with impaired bADL, and pain at the lumbar spine was specifically associated with sleeping problems. Reduced exercise played a role in both lumbar spine and hip pain, but not in knee pain.

**Table 4:** Association of pain at the 3 most frequent sites (knee, lumbar spine, hip) with patient characteristics ± in a multivariate stepwise forward logistic regression analysis

		95 % CI		
	OR	Lower	Upper	*p* value
frontal knee (n = 74)				
no. pain sites	1.72	1.43	2.06	<0.001
bADL	3.79	2.01	7.16	<0.001
lumbar spine (n = 73)				
no. pain sites	1.53	1.30	1.81	<0.001
sleeping disorder	1.94	1.07	3.52	0.03
not enough exercise	1.99	1.09	3.62	0.03
frontal hip (n = 30)				
no. pain sites	1.33	1.12	1.58	0.001
not enough exercise	2.27	1.04	4.95	0.04

±14 parameters assessed as part of the comprehensive geriatric assessment

*bADL* activities of daily living, *CI* confidence interval

### Patients’ and physicians’ priorities

Of the 219 patients suffering from pain at any site, 80.3 % (n = 175) rated their pain as moderate to severe and/or restricting their daily activities. These patients were then asked to rate the importance of pain along with their other health problems. 130 of these 175 patients rated their pain as “important” (74.3 %). Their physicians also rated the importance, once for their medical care and once when putting themselves in the patients’ position. The physicians classified the pain as important for their care in 146 patients (83.4 %). They presumed that the pain was important for 154 patients (88.0 %). Doctors and patients agreed on the importance of the pain in 69.1 % (121/175) of cases (κ *=* 0.09) when doctors considered the importance for their care. The agreement was slightly higher when doctors put themselves in the patients’ position: 73.7 % (129/175) agreement, κ *=* 0.17. Overall, physicians overrated the importance of pain in both situations compared to the patients’ assessment.

## Discussion

### Overview of findings

Our geriatric assessment in a general practice sample revealed that 3 out of 4 older patients suffered from pain in the preceding four weeks. Most pain-sufferers reported that their pain was at least moderate or restricted their daily activities. Usually multiple locations were affected. We also found that a specific location of pain determined the pattern of concurring health problems. However, due to the study design these associations cannot be interpreted as causal. GPs often misconceived the pain experiences of their patients. They tended to overrate the importance of pain.

### Comparison with the literature

Although specific pain, such as back pain, is commonly investigated, little attention has been paid to the presence of yet “unspecified” pain, which may include different pains or multiple locations. Nonetheless, this is relevant to GPs who initially deal with non-specific or multiple pain presentations on a daily basis. Prevalence rates of such unspecified pain in a German population survey are quite high with 77 % of people aged 65 years and over reporting pain within the last four weeks [17]. Similar figures was found in the North-Staffordshire study, [18]. However, when pain was assessed as a reason for encounter, it occurred in only 28 % of patients in a Stockholm surgery or in 21 % of patients 65 + years in a German general practice [2, 19]. Using the approach of a geriatric assessment in general practice, we obtained high prevalence figures consistent with population data. Considering the much lower prevalence rates for pain presented in practice consultations, our findings support the assumption that indeed underreporting takes place [3].

Multisite pain is a predominant feature in older people. Our assessment revealed that among the subset of patients with pain, two thirds located symptoms at two or more sites. This finding is comparable with US population studies showing prevalence figures of 63 % [20] and 75 % [20] for people experiencing pain aged 65 years and more. Multisite pain has an aggravating independent effect on disability and social functioning [12, 20, 21]. Therefore it is worthwhile for GPs to actively seek for multisite pain and not only focus on the main pain site.

The impact of pain has been investigated in several large population studies. There is evidence that sleep disorders, functional disabilities, falls, depression and low levels of physical activity are related to pain [22–27]. Less clear are associations of specific pain locations with these related problems, as has been shown for falls and activities of daily living [7, 28]. Our analysis on three pain sites showed differential associations, i.e. sleep disorders with lower back pain, restrictions of daily activities with knee pain, and insufficient exercise with hip or lower back pain.

*Sleep disorders* occurred in combination with backache, limb pain and headaches in a large European adult population survey [29]. In an older population survey in Boston the only single pain sites independently related to sleep disorders were the hand and the shoulder. However, multisite pain that included sites of back, hip and knee were associated with sleep problems [22]. *Restrictions of daily activities* were shown to be associated with different pain locations, such as hip, back, knee, feet and hand [30] but also just with hip pain [31]. In our study, we found an association between activities of daily living and lower back, hip and knee pain, but only the association with the knee site remained after adjustments. Our ADL questionnaires included stair climbing, as this is particularly relevant for many older people in Germany. However, well established ADL questionnaires, such as from Lawton and Brody [32], do not include this item. As regards “*not enough exercise*” we found an association with hip and lower back pain, but not with knee pain after adjustments. A Dutch population based study found a U-shaped relationship between back pain and exercise with very little and very much exercise being related to pain [33]. Moreover, physical inactivity has been related to hip pain [34] and severe but not moderate knee pain [35].

We found no significant associations of pain with f*alls and fractures* for any of the three pain sites (lower back, hip, and knee). However, in our study, the occurrence of falls was very low so that inference statistics show no significant differences between groups. The Women’s Health and Aging Study reported an association merely in women with widespread pain or lower extremity pain [7]. We also did not find any particular association between pain location and *depression* after adjustments. In population studies [36, 37] depressive symptoms were not specifically associated with any particular pain location, but to the severity of pain. Considering these heterogeneous findings, we can reasonably assume that pain location is just one factor amongst a variety of other pain and health related factors, attitudes and behaviours, that mediate or impact on daily functioning and mood [38].

The importance of pain is usually not part of a pain assessment, in which intensity, functional restrictions and distress are typical measures. It has been argued that information about the personal importance should be included in pain assessments to identify patient needs and priorities more comprehensively [39]. Indeed, GPs should be aware of patient priorities to optimize health care goals and treatments with their patients. The assumption that pain severity alone is a sufficient marker for treatment priority has been found to be inaccurate [40]. In the present study we deployed a simple question of importance as a priority check. Three quarters of the patients who triggered in the geriatric assessment because of moderate to severe pain or interference in daily activities assessed their pain as important. However, one quarter did not find their pain important despite it being severe or disabling. This may be due to coping with the problem – as a result of weighing-up co-existing health problems in relation to each other. In contrast, GPs overrated the importance of pain for the affected patients; and the dyadic doctor-patient agreement on the importance of pain was low. The few previous findings indicate that doctors tend to underrate the severity of pain and its effect on their patients [41, 42]. The seeming discrepancy of agreement that occurs for pain severity and perceived importance also suggests that importance is an additional dimension of pain perception. It may therefore be a valuable addition in the decision-making on pain treatments [43].

### Strengths and weaknesses

This is a post-hoc analysis of a cross-sectional study on pain locations and its associations in an older general practice population. One novel aspect of our study is the sampling technique as each patient underwent a geriatric assessment that included pain avoiding the presence of pain as key question for inclusion of patients. The prevalence of pain therefore exceeds that of other general practice studies, which rely on more self-motivated patient reports, indicating the presence of underreporting. Also, pain assessment as part of an overall geriatric assessment facilitates a comprehensive association with other common health related conditions and everyday problems of older patients in primary care. Finally, to our knowledge the dyadic comparison of the importance of pain between doctors and patients has not been investigated before.

One of the limitations of the current study is that it was a cross-sectional analysis portraying the concurrent relationship between pain and other health related conditions. Therefore the findings cannot contribute towards clarifying the complex causal relationships of pain locations and health related problems. The study does not provide any information on other health conditions or patient characteristics than those collected as part of the geriatric assessment. Further data might have enriched our analyses, e.g. BMI or coping skills. Moreover, due to our geriatric assessment procedure, the association of pain with other health related conditions was only possible for the (large) subgroup of patients with more severe pain (trigger was pain assessed as moderate to severe or impeding daily activities). Finally results of the current study are not easily comparable with other findings for very different reasons, e.g.: the level of care, inclusion criteria, definition and measurement of pain, and inclusion of different factors to be associated with pain.

## Conclusions

Our findings suggest the necessity of a pro-active doctor-driven diagnostic approach for older patients in general practice as pain is seemingly not reported by all pain sufferers in this age group. GPs should also pay particular attention to multisite pain, as only a minority of older patients have single site pain, even if just the main pain site is mentioned. The findings also point towards a considerable discordance of patients’ and doctors’ views on the importance of pain. When decisions on pain treatment emerge, GPs should also consider the patient perception on importance and establish treatment preferences within a wide range of treatments that co-exist in the presence of multiple health problems. As different pain locations are associated with different limitations in quality of life, complex rehabilitation and support might be required beyond the prescription of universal “pain killers”.
